# Macro-Aspartate Aminotransferase: A Benign Cause of Abnormal Laboratory Findings

**DOI:** 10.7759/cureus.23841

**Published:** 2022-04-05

**Authors:** Nikhil Reddy, Fadi S Odish, Sangeeta Agrawal

**Affiliations:** 1 Internal Medicine, Boonshoft School of Medicine, Dayton, USA; 2 Gastroenterology, Dayton Veterans Affairs Medical Center, Wright State University, Dayton, USA

**Keywords:** liver, ast (aspartate aminotransferase), peg precipitation, macroenzymes, macroast

## Abstract

Isolated, persistent elevations of aspartate aminotransferase (AST) without plausible explanation may lead to diagnostic confusion. Macro-enzyme aspartate aminotransferase (macro-AST) is an uncommon and benign cause of persistently elevated AST levels in the absence of other diseases. We present a case of macro-AST in an otherwise healthy adult male which was confirmed by polyethylene glycol (PEG) precipitation.

## Introduction

Aspartate aminotransferase (AST) elevations have been associated with cardiac, liver, and musculoskeletal disease [1]. Persistent elevations of AST without plausible explanation may lead to diagnostic confusion. Macro-enzyme aspartate aminotransferase (macro-AST) is an uncommon and benign cause of isolated, persistently elevated AST levels. This condition occurs due to the formation of macro complexes from either self-polymerization or association with other serum components leading to decreased renal clearance and isolated elevation of AST [2]. This phenomenon is uncommon with few reported case reports and requires specialized testing. Polyethylene glycol (PEG) precipitation testing is one known modality which can account for macro-AST presence [1]. Awareness of the entity and high clinical suspicion can help avoid extensive and invasive testing in the appropriate clinical setting. In this case, we describe a case of macro-AST in an otherwise healthy adult male.

## Case presentation

A healthy 48-year-old Caucasian male was referred to the hepatology clinic for persistently elevated AST levels on November 9, 2021. The patient had no clinical symptoms and a physical exam was unremarkable. He denied any alcohol use or a family history of liver disease. His only medication was an albuterol inhaler. At the time of referral, his AST was 482 U/L (normal 9-45) with no other significant laboratory abnormalities. Further chart review revealed the patient had an elevated AST level dating back to 2011 with a progressive rise in AST level (Table 1). Additional work-up included viral hepatitis, autoimmune hepatitis ceruloplasmin, alpha-1-antitrypsin, ferritin level, and creatinine kinase which were all unremarkable. A right upper quadrant ultrasound showed mild steatosis. Given the unremarkable work-up and benign chronicity of the elevated AST, macro-AST was suspected. PEG precipitation testing was then performed which was sent to a specialized laboratory. This testing revealed a serum AST activity level of 405 U/L and a post-PEG precipitation level of 33 U/L, corresponding to a 92% activity of macro-AST (reference level >80% indicated presence of macro-AST). A liver biopsy was not performed due to patient preference. 

**Table 1:** Chronological aspartate aminotransferase (AST) levels

Date	AST (U/L)
12/08/11	212
02/05/13	225
03/04/15	432
05/26/16	516
03/04/19	426
10/26/20	425
10/26/21	518
11/09/21	482

## Discussion

Macroenzymes are serum enzymes formed either through self-polymerization or through association with other serum molecules. These macroenzymes have a larger molecular weight, resulting in a reduced renal clearance [2]. This reduction leads to accumulation of the macroenzyme in the blood and correspondingly false elevations on standard testing [2]. Macro-AST has been described in a few case reports and is an uncommon, benign etiology of isolated elevation in AST levels. Prevalence appears to be lower than other macro-enzyme forms with macro-amylasemia being the most common [3]. Macro-AST can be associated with immune-related and neoplastic disorders but is most often benign [4]. Providers should have a high clinical suspicion in the appropriate setting, but other causes of elevated AST levels need to be considered and ruled out. Our clinical suspicion was driven by the significant isolated elevation in AST levels and chronic duration of the elevated enzyme without other evidence of chronic liver diseases. PEG-precipitation has been found to be a reliable method of detection, although alternative methods are available such as protein electrophoresis and gel filtration chromatography [1]. Once diagnosed, many patients do not require further work-up or evaluation. However, given an association with immunologically-related conditions and neoplastic disease, providers may consider either a rheumatologic or oncologic assessment in the appropriate context, such as weight loss or other symptoms.

## Conclusions

This case overall brings attention to a rare but benign cause of isolated AST elevation which can be a challenging clinical scenario. Appropriate evaluation with macroenzyme assays may avoid unnecessary procedures or extensive testing. As demonstrated by our patient, AST levels may remain elevated for years to decades. As a result, continued documentation is necessary to avoid the use of further medical resources for diagnostic work-up. In addition, patients should be educated about their condition and most should be reassured that further evaluation is unnecessary. In the appropriate clinical context, however, rheumatologic or oncologic assessment may be considered. 
